# Network pharmacology unveils the active components and potential mechanism of traditional efficacy of Mugua

**DOI:** 10.1097/MD.0000000000041028

**Published:** 2024-12-20

**Authors:** Yonggang Wu, Shijun Yue

**Affiliations:** a School of Marxism, Shaanxi University of Chinese Medicine, Xi’an, China; b School of Pharmacy, International Joint Research Center on Resource Utilization and Quality Evaluation of Traditional Chinese Medicine of Hebei Province, Hebei University of Chinese Medicine, Shijiazhuang, China.

**Keywords:** anti-inflammatory, molecular docking, Mugua, network pharmacology, targets, traditional efficacy

## Abstract

Mugua is a Chinese herbal medicine derived from the dried mature fruit of *Chaenomeles speciosa* (Sweet) Nakai. This study aimed to dissect the active ingredients and mechanism of Mugua. In the present study, the active components of Mugua were collected and screened through databases combined with UPLC-Q/TOF-MS based qualitative analysis and literature mining, and their potential disease targets were predicted. Then, a network relationship diagram of “component-target-disease-efficacy” was constructed. Moreover, the key active components and core targets were analyzed by molecular docking and in vitro anti-inflammatory assays. The traditional efficacy of Mugua mainly corresponded to 4 diseases, namely, rheumatoid arthritis, diarrhea, edema, and emesis. After screening and comparison, it was found that IL-1β, IL-6, TNF, and epidermal growth factor receptor (EGFR) were the shared inflammatory targets of the 4 diseases. Gene ontology and Kyoto Encyclopedia of Genes and Genomes pathway enrichment results showed that these targets were involved mainly in inflammatory responses and inflammation-related pathways, such as rheumatoid arthritis pathway and TNF signaling pathway. Network topology analysis revealed that succinic acid, cinnamic acid, citric acid, caffeic acid, gallic acid, ursolic acid, malic acid, betulinic acid, and oleanolic acid were the key active components, while IL-1β, IL-6, TNF, and EGFR were the shared core targets of these 4 diseases. These results suggested that Mugua could exert traditional efficacy through multi-component and multi-target synergistic mechanisms. Molecular docking results showed that all key active ingredients could autonomously bind to the shared core targets, and the in vitro anti-inflammatory results further confirmed that all the key active components had good anti-inflammatory activities. The present study found that Mugua mainly intervened in the inflammatory response and pathways by acting on key active components and core targets to exert traditional efficacy, providing a theoretical basis for further in-depth research.

## 1. Introduction

Mugua is a Chinese herbal medicine derived from the dried mature fruit of *Chaenomeles speciosa* (Sweet) Nakai. It has a warm nature and a sour taste and is known for its effects on soothing muscles and activating collaterals, harmonizing the stomach, and transforming dampness. It is used to treat symptoms such as damp-bi syndrome with contraction and spasm, sore and heavy pain in the waist and knee joints, summer-damp vomiting and diarrhea, muscle cramps, and edema of the legs and feet.^[1]^ Mugua contains diverse chemical components, such as triterpenoids, flavonoids, phenolic acids, amino acids, and sugars.^[2]^ Modern pharmacological studies have shown that Mugua has anti-inflammatory, analgesic, antimicrobial, antioxidant, immune regulatory, and gastrointestinal protective effects,^[3]^ which coincide with its traditional efficacy. However, the chemical composition of Mugua is complex, and existing research has not systematically clarified the active components and molecular mechanisms by which it exerts its traditional efficacy. Network pharmacology explains the complex biological network relationships between drugs, diseases, components, and targets at the systemic level, revealing the mechanisms by which drugs exert their effects.^[4]^ This study used network pharmacology to collect and screen the active components of Mugua, predict its potential disease targets, perform bioinformatics analysis, and construct a network relationship diagram of “component-target-disease-efficacy” to explore the underlying mechanism by which Mugua exerts traditional efficacy. Finally, the key active components and shared core targets were analyzed by molecular docking and in vitro anti-inflammatory activity evaluation, providing a theoretical basis for further in-depth research.

## 2. Materials and methods

### 2.1. Preparation of Mugua extract

Mugua powder (0.5 g) was accurately weighed, and placed in a conical flask, where 25 mL of methanol was accurately added. The flask was sealed tightly, weighed, and sonicated (250 W, 40 kHz) for 20 minutes. The flask was allowed to cool and reweighed, and fresh methanol was added to compensate for weight loss. The extract was obtained and further diluted 100 times, filtered through a 0.22 mm membrane, and centrifuged (13,000 rpm, 10 minutes, 4 °C). The supernatant was collected for UPLC-Q/TOF-MS analysis.

### 2.2. UPLC-Q/TOF-MS analysis

The ACQUITY UPLC system was equipped with a waters acquity BEH C18 column (100 mm × 2.1 mm, 1.7 mm), and the auto-sampler temperature was set at 4 °C. The column temperature was maintained at 40 °C, and the mobile phase consisted of 0.1% formic acid in water (A) and acetonitrile (B), with a flow rate of 0.3 mL/min. The gradient elution program was as follows: 0 to 0.5 minutes, 10% B; 0.5 to 2 minutes, 10% to 25% B; 2 to 15 minutes, 25% to 98% B; 15 to 16 minutes, 98% B; 16 to 18 minutes, 98% to 10% B; and 18 to 20 minutes, 10% B. The injection volume was 2 mL.

The Xevo G2 QTOF mass spectrometer was operated in both positive and negative modes, using full scan mode. The electrospray ionization (ESI) conditions used were as follows: capillary voltage of 3 kV, sample cone temperature of 55 °C, source temperature of 100 °C, desolvation gas temperature of 100 °C, cone gas flow rate of 50 L/h, desolvation gas (N_2_) flow rate of 800 L/h, and mass range of *m*/*z* 50 to 1200 Da.

All data acquisition and analysis were controlled using Waters MassLynx v4.1 software. Chemical components were identified based on their retention times and secondary fragments in both positive and negative ion modes, compared with relevant literature data, and confirmed in conjunction with extracted ion chromatograms.

### 2.3. Collection and screening of the main active components of Mugua

The chemical components of Mugua were collected using the traditional Chinese medicine system pharmacology database and analysis platform (TCMSP, https://www.tcmsp-e.com/#/home) and supplemented by searching the keywords “mugua” or “*Chaenomeles speciosa*” on the China National Knowledge Infrastructure, Google Scholar, and PubMed. The oral bioavailability (OB) and drug-likeness (DL) values of the chemical components of Mugua were collected and organized through the TCMSP database. An OB value ≥ 30% and a DL value ≥ 0.18 were set as the thresholds for the selection of active components. Finally, the reported active components and the screened active components were integrated and included in subsequent analysis.

### 2.4. Prediction and analysis of potential targets

All the active components of Mugua were input into the GeneCards (https://www.genecards.org/) and CTD (http://ctdbase.org/) to obtain their related targets and remove duplicate values. The obtained targets were imported into the UniProt database (http://www.uniprot.org/) to obtain the corresponding abbreviations. Then, the diseases directly related to the traditional efficacy of Mugua were determined through the SymMap database (https://www.symmap.org/) and a literature review, and the disease names were input into the GeneCards database to obtain the corresponding targets of the diseases. Finally, the intersection of the related targets of the active components and the diseases were obtained as the potential targets of Mugua.

### 2.5. Construction of the protein-protein interaction network and screening of potential core targets

The potential targets of Mugua were imported into the STRING database (https://www.string-db.org/), with a medium confidence level (0.400), a maximum interaction number of no more than 5, and no network clustering to obtain the interaction relationships between the targets. Then, they were imported into the Cytoscape 3.6.1 software to construct a protein-protein interaction (PPI) network. The potential targets were imported into the VarElect website (https://ve.genecards.org) to obtain the association scores of the targets and the diseases. Finally, the top ten (the degree value) targets in the PPI network and the top ten targets (the association degree score) were taken as the intersection to obtain the potential core targets of the diseases.

### 2.6. Gene ontology enrichment and Kyoto Encyclopedia of Genes and Genomes pathway analysis

The potential targets of Mugua were imported into the DAVID database (https://davidbioinformatics.nih.gov/), and the species were set as “*Homo sapiens*” to perform GO functional enrichment and KEGG pathway enrichment analyses. The GO functional enrichment analysis included biological process (BP), molecular function (MF), and cellular component (CC). When performing KEGG pathway analysis, *P* < .05 was set, and the pathways were sorted by *P*-value from small to large. Then, the top 10 results for all GO and KEGG enrichment analyses were selected and visualized.

### 2.7. Construction of Mugua network relationship diagram

The relationships between the active components of Mugua and their potential targets and the corresponding diseases and effects were organized and imported into Cytoscape 3.6.1 software to construct a network. The network analyzer function was used to perform topological analysis on this network, and the degree values of each node were obtained. Based on the degree values and types of nodes, the size and color of the nodes were adjusted to create the “component-target-disease-effect” network diagram of Mugua.

### 2.8. Molecular docking

To verify the reliability of the network pharmacology analysis results, the top 9 active components with high degree values and the shared core targets for the treatment of 4 diseases by Mugua were subjected to molecular docking using AutoDock Vina software. The 3-dimensional structures of the shared core targets and the chemical structures of the active components were obtained from the RCSB PDB (http://www.rcsb.org/) and the ZINC database (https://zinc.docking.org/), respectively. The targets and component molecules were processed using AutoDock Tools, and the docking active sites were obtained using the Autogrid plugin for molecular docking.

### 2.9. In vitro anti-inflammatory assay

An in vitro anti-inflammatory assay was designed for the 9 active components with high degree values in the network. Succinic acid, cinnamic acid, citric acid, caffeic acid, gallic acid, ursolic acid, malic acid, betulinic acid, and oleanolic acid were purchased from Shanghai Yuanye Bio-Technology Co., Ltd. BV-2 mouse microglial cells were purchased from the American Type Culture Collection and cultured in the logarithmic growth phase. The undifferentiated cells were seeded at 3 × 10^4^/well in 48-well plates and treated with lipopolysaccharide (LPS, 1 μg/mL, cultured for 6 hours), compound + LPS (compound pretreatment 30 minutes before LPS stimulation), or other groups as needed. After 24 hours, the supernatants were collected, and the levels of IL-1β, IL-6, and TNF-α in the supernatants were detected using ELISA according to the instructions of the ELISA kits purchased from Shanghai Enzyme-Linked Biological Technology Co., Ltd. The nitric oxide (NO) concentration was detected using the NO chemical method kit from Nanjing Jiancheng Bioengineering Research Institute according to the instructions.

### 2.10. Ethical approval

The current analysis does not require ethical approval, because all procedures performed in the present study did not involve human participants or animals.

### 2.11. Statistical analysis

Data were expressed as mean ± standard error (SD). GraphPad Prism 8.0 and IBM SPSS Statistics 24 software were used for statistical analysis. The *t*-test was used for comparisons between 2 groups, and *P* < .05 was considered statistically significant.

## 3. Results

### 3.1. Qualitative identification of components in Mugua

**Figure 1.**
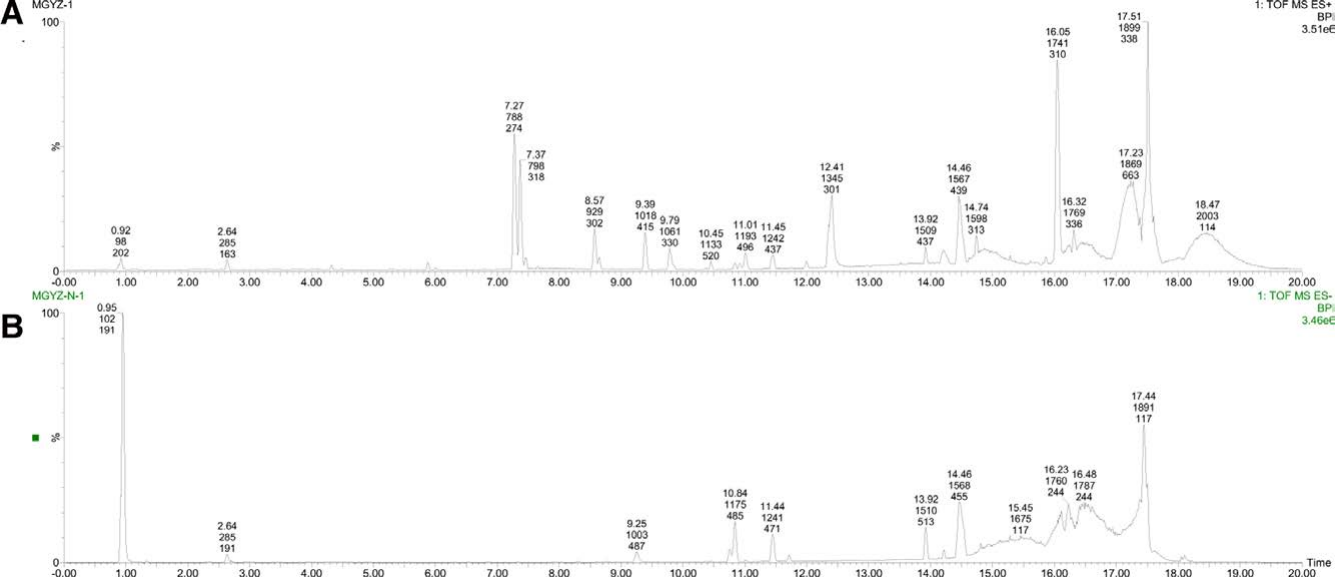
The base peak ion chromatograms of Mugua samples in positive (A) and negative (B) ion modes.

UPLC-Q/TOF-MS was used to analyze the components in Mugua, resulting in the base peak ion chromatograms in both positive and negative ion modes (Fig. 1). By comparing the accurate mass-to-charge ratios with data from the literature and databases, a total of 13 compounds were identified, with the mass spectrometry information provided in Table 1.

**Table 1:** Qualitative identification of components in Mugua.

No.	*t*_R_/min	Component	Molecular formula	Theoretical value of *m*/*z*	Actual value of *m*/*z*	Error (ppm)	Fragment ion
1	1.329	Shikimic acid	C_7_H_10_O_5_	173.045 0	173.0 458	4.623	137.021 1, 111.010 5
2	2.621	Quinic acid	C_7_H_12_O_6_	191.055 6	191.0 565	4.711	174.953 5
3	2.630	Chlorogenic acid	C_16_H_18_O_9_	353.087 3	353.0 888	4.248	173.040 0, 161.025 3
4	2.699	(−)-Catechin	C_15_H_14_O_6_	289.071 2	289.0 708	–1.384	245.079 8, 179.039 9
5	2.707	L-epicatechin	C_15_H_14_O_6_	289.071 2	289.0 708	–1.384	179.953 5, 135.175 9
6	3.039	Caffeic acid	C_9_H_8_O_4_	179.034 4	179.0 341	–1.676	135.043 8
7	3.344	Rutin	C_27_H_30_O_16_	609.145 6	609.1 476	3.283	300.132 0, 271.214 5
8	3.494	Hyperoside	C_21_H_20_O_12_	463.087 7	463.089 1	3.023	300.018 5, 271.228 9
9	12.379	Cinnamic acid	C_9_H_8_O_2_	149.060 3	149.0 61 7	9.392	140.028 2, 100.995 4
10	14.220	Ursolic acid	C_30_H_48_O_3_	455.352 5	455.355 2	5.929	407.209 7
11	14.464	Oleanic acid	C_30_H_48_O_3_	455.352 5	455.355 2	5.929	407.209 7
12	14.717	Betulinic acid	C_30_H_48_O_3_	455.352 5	455.355 2	5.929	407.209 7
13	16.305	3-Acetylursolic acid	C_32_H_50_O_4_	497.363 1	497.361 8	–2.614	437.355 4, 401.096 3

### 3.2. Screening of active components of Mugua

A total of 95 chemical components of Mugua were collected through the TCMSP database and relevant literature. After screening based on the criteria of OB value ≥ 30% and DL value ≥ 0.18, and supplementing chemical components with related activity reports, 25 active components were finally identified, including 6 triterpenoid components, 6 flavonoid components, and 13 organic acid components (Table 2).

**Table 2:** Active components of Mugua.

No.	Chemical name	Oral bioavailability (%)	Drug-likeness
1	Oleanic acid	29.02	0.76
2	Ursolic acid	16.77	0.75
3	3-Acetylursolic acid	15.23	0.70
4	Betulin	15.48	0.78
5	Betulinic acid	55.38	0.78
6	Corosolic acid	15.86	0.74
7	Rutin	3.20	0.68
8	Hyperoside	6.94	0.77
9	(−)-Catechin	49.68	0.24
10	Quercetin	46.43	0.28
11	Kaempferol	41.88	0.24
12	L-epicatechin	28.93	0.24
13	Kojic acid	48.70	0.03
14	D(−)-Tartaric acid	45.27	0.02
15	Succinic acid	29.62	0.01
16	Malic acid	68.62	0.02
17	Cinnamic acid	19.68	0.03
18	Citric acid	56.22	0.05
19	Quinic acid	63.53	0.06
20	Shikimic acid	46.24	0.04
21	Gallic acid	31.69	0.04
22	Chlorogenic acid	11.93	0.33
23	Protocatechuic acid	25.37	0.04
24	Caffeic acid	25.76	0.05
25	Arachidonic acid	45.57	0.20

### 3.3. Prediction of potential targets of Mugua

**Figure 2.**
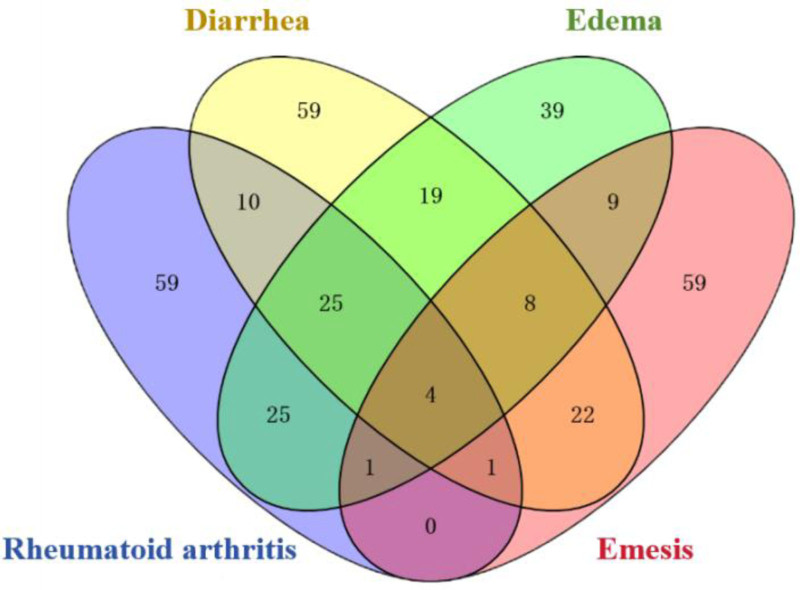
Venn diagram of the intersection of targets for each disease affected by Mugua.

A total of 966 target points of the active components of Mugua were collected through the GeneCards and CTD databases. According to the SymMap database and literature review, Mugua traditional efficacy corresponds to 4 diseases: rheumatoid arthritis, diarrhea, edema, and emesis. Using GeneCards, 326 related targets for rheumatoid arthritis, 347 for diarrhea, 307 for edema, and 256 for emesis were collected. The intersection of disease targets and component targets was compared, and 125 potential targets for Mugua in the treatment of rheumatoid arthritis, 148 for diarrhea, 130 for edema, and 104 for emesis were identified. Further intersection comparison revealed the shared core targets of Mugua treatment for the 4 diseases: IL-1β, IL-6, TNF, and epidermal growth factor receptor (EGFR), as shown in Figure 2. IL-1β is a pro-inflammatory cytokine that plays a key role in the inflammatory response.^[5]^ IL-6 is also a pro-inflammatory cytokine produced at the site of inflammation and induces the transcription of its receptor to participate in the inflammatory response.^[6]^ TNF is a pleiotropic cytokine with a key role in inflammation that can activate NF-κB, leading to inflammation.^[7]^ VEGF is a mediator of angiogenesis and inflammation, and VEGF is synergistically induced by IL-1β, TNF, and IL-6, which play a role in the inflammatory response and angiogenesis.^[8]^

### 3.4. Protein–protein interaction network analysis

**Figure 3.**
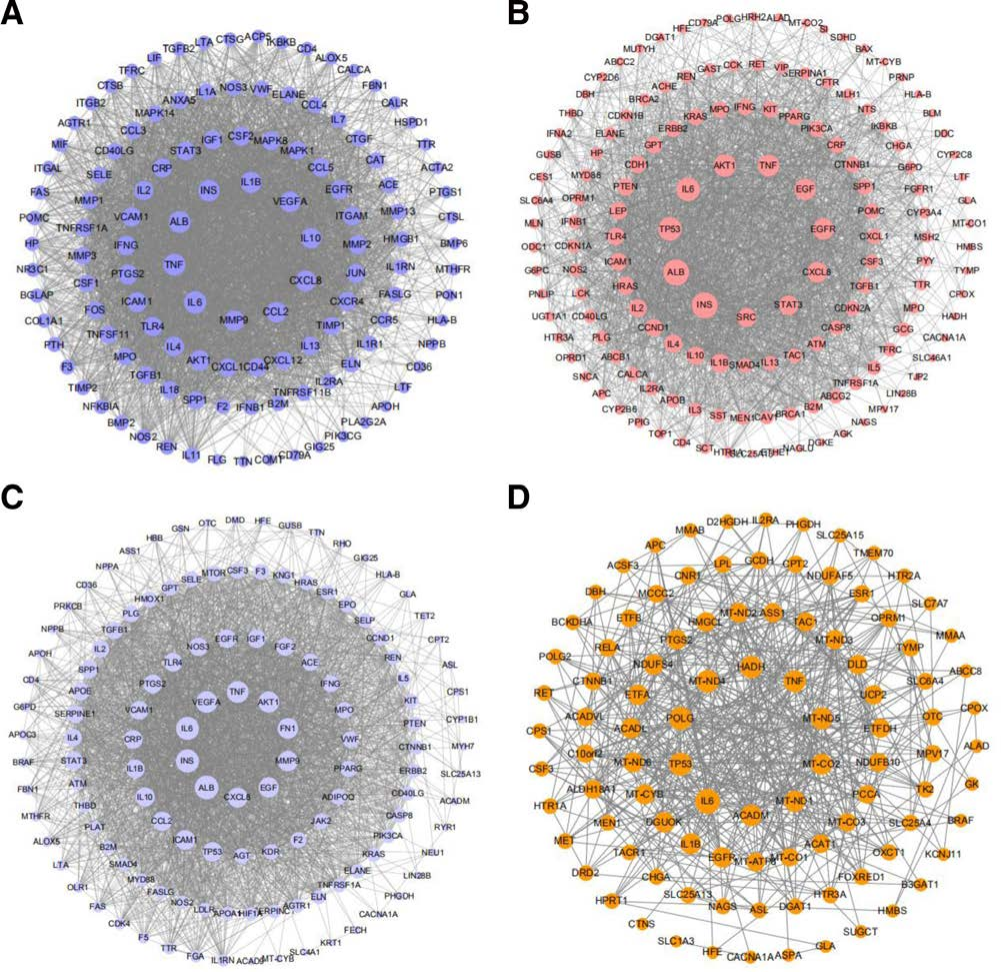
PPI network diagrams of Mugua targets for rheumatoid arthritis (A), diarrhea (B), edema (C), and emesis (D). PPI = protein–protein interaction.

The targets were imported into the VarElect database to obtain the association scores of the targets and the diseases. The top ten targets with association scores were presented in Table 3. The top ten targets in terms of degree value and association degree score were intersected to obtain the targets with high degree values and close relationships with the diseases. Figure 3A shows the PPI network diagram of Mugua treatment for rheumatoid arthritis, which involves 122 nodes and 3037 edges. The potential core targets for Mugua treatment of rheumatoid arthritis are IL-6, TNF, IL-1β, and IL-10. Figure 3B shows the PPI network diagram of Mugua treatment for diarrhea, which involves 147 nodes and 2181 edges. The potential core targets for Mugua treatment of diarrhea are IL-6, TNF, and EGFR. Figure 3C shows the PPI network diagram of Mugua treatment for edema, which involves 129 nodes and 2536 edges. The potential core targets for Mugua treatment of edema are serum albumin (ALB), IL-6, vascular endothelial growth factor A (VEGFA), and TNF. Figure 3D shows the PPI network diagram of Mugua treatment for emesis, which involves 99 nodes and 503 edges. The potential core targets for Mugua treatment of emesis are IL-6, POLG (mitochondrial DNA polymerase catalytic subunit), and IL-1β. The analysis revealed that IL-1β, IL-6, TNF, and other inflammation-related targets occupy important positions in the network, indicating that these targets may play important roles in the treatment of diseases with Mugua.

**Table 3:** Targets and disease association scores.

Disease	Target	Association score	Disease	Target	Association score
Rheumatoid arthritis	IL-6	62.51	Emesis	HTR3A	8.57
	IL-10	62.33		TACR1	4.49
	TNF	53.51		TYMP	4.34
	HLA-B	43.09		POLG	4.34
	MIF	39.29		TAC1	3.16
	LTA	36.28		DRD2	2.39
	IL1β	33.85		CNR1	2.16
	IL2RA	32.24		CHGA	1.80
	CRP	31.87		IL-6	0.43
	TNFRSF1A	28.49		IL1β	0.36
Diarrhea	AGK	14.91	Edema	PLG	18.50
	EGFR	13.73		ALB	16.70
	IL-10	13.09		SLC4A1	15.65
	CFTR	13.00		VEGFA	12.94
	SI	12.37		IL-6	12.34
	ETHE1	12.31		ACE	11.63
	TGFB1	11.89		KNG1	11.19
	CD40LG	11.87		TNF	10.51
	IL-6	11.65		RYR1	10.40
	TNF	11.50		NEU1	10.32

### 3.5. GO enrichment analysis and KEGG pathway analysis

**Figure 4.**
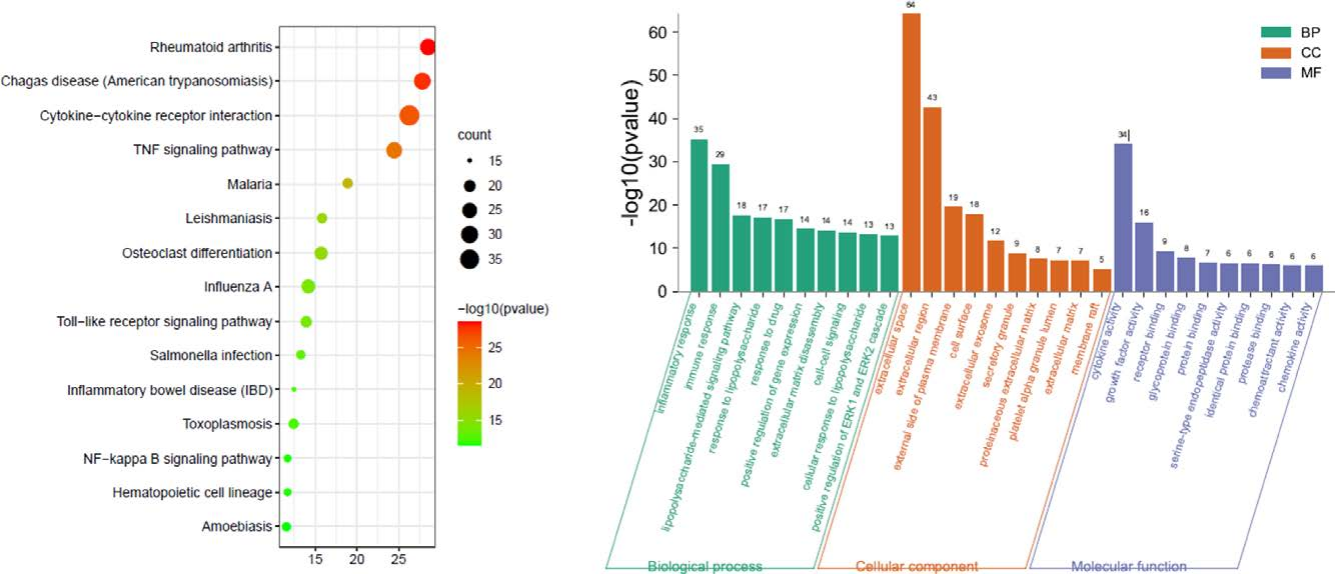
Kyoto Encyclopedia of Genes and Genomes (KEGG) pathway and gene ontology (GO) function enrichment analysis of Mugua in the treatment of rheumatoid arthritis.

The GO enrichment results for the treatment of rheumatoid arthritis with Mugua were shown in Figure 4. The BPs involved in Mugua treatment of rheumatoid arthritis mainly include the inflammatory response, immune response, and lipopolysaccharide-mediated signaling pathway. The target proteins are mainly involved in the construction of the extracellular region, extracellular space, outside of the plasma membrane, and cell surface in terms of CCs. In terms of MFs, they are mainly involved in regulating cytokine activity, growth factor activity, receptor binding regulation, and protein binding. The enriched KEGG pathways were mainly involved in rheumatoid arthritis, cytokine-cytokine receptor interaction, TNF signaling pathway, osteoclast differentiation, and NF-κB signaling pathway.

**Figure 5.**
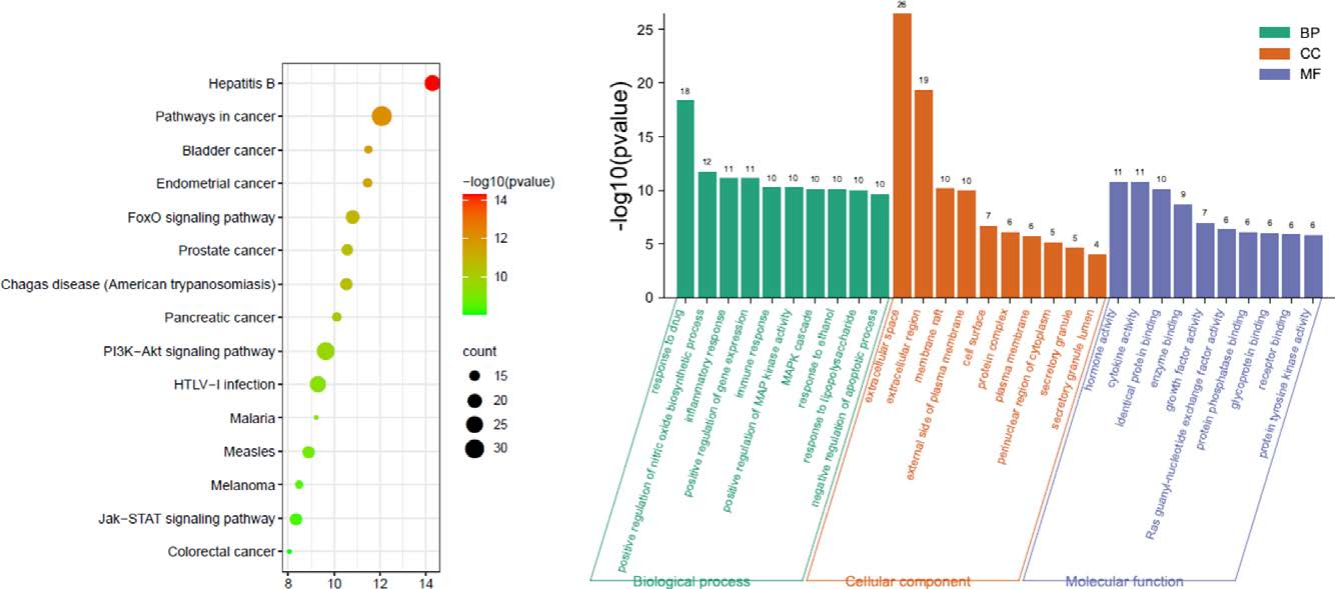
KEGG pathway and GO function enrichment analysis of Mugua in the treatment of diarrhea. GO = gene ontology, KEGG = Kyoto Encyclopedia of Genes and Genomes.

The GO enrichment results for the treatment of diarrhea with Mugua were shown in Figure 5. The BPs involved in Mugua treatment of diarrhea mainly include drug response, positive regulation of NO biosynthetic process, inflammatory response, positive regulation of gene expression, and immune response. The target proteins are mainly involved in the construction of extracellular regions, plasma membrane exterior, cell surface, protein complex, and extracellular matrix in terms of CCs. In terms of MFs, they are mainly involved in regulating hormone activity, cytokine activity, growth factor activity, regulation of the same protein binding, and enzyme binding. The enriched KEGG pathways included mainly PI3K-Akt signaling pathway, FoxO signaling pathway, and cancer-related pathways.

**Figure 6.**
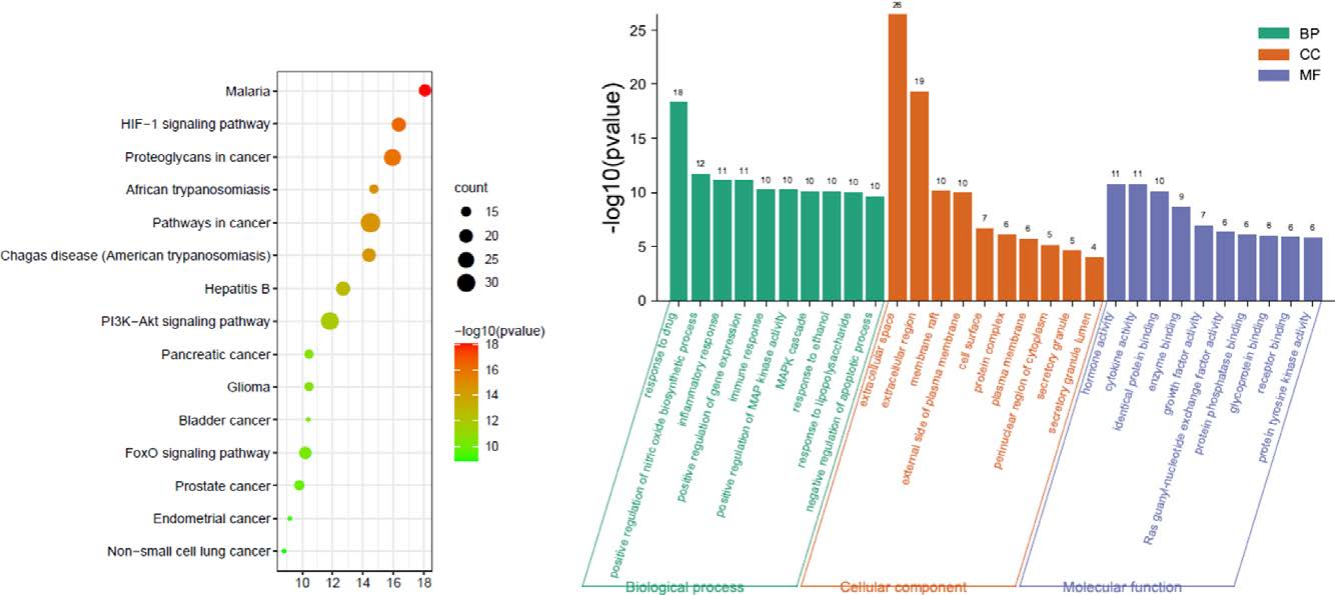
KEGG pathway and GO function enrichment analysis of Mugua in the treatment of edema. GO = gene ontology, KEGG = Kyoto Encyclopedia of Genes and Genomes.

The GO enrichment results for the treatment of edema with Mugua were shown in Figure 6. The BPs involved in Mugua treatment of edema mainly include drug response, positive regulation of NO biosynthetic process, inflammatory response, positive regulation of gene expression, and immune response. The target proteins are mainly involved in the construction of membrane rafts, plasma membrane exterior, cell surface, protein complex, and secretory granules in terms of CCs. In terms of MFs, they are mainly involved in regulating hormone activity, cytokine activity, same protein binding, enzyme binding, and Ras guanyl-nucleotide exchange factor activity. The enriched KEGG pathways included mainly HIF-1 signaling pathway, cancer-related pathways, PI3K-Akt signaling pathway, and FoxO signaling pathway.

**Figure 7.**
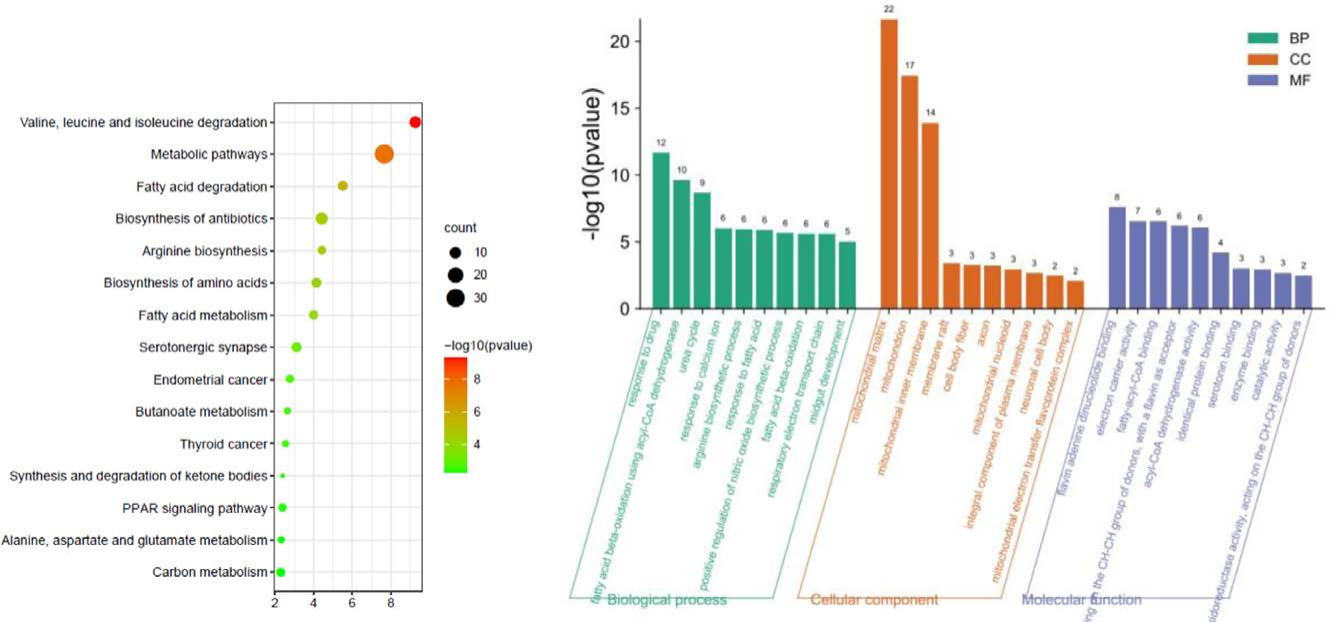
KEGG pathway and GO function enrichment analysis of Mugua in the treatment of emesis. GO = gene ontology, KEGG = Kyoto Encyclopedia of Genes and Genomes.

The GO enrichment results for the treatment of emesis with Mugua were shown in Figure 7. The BPs involved in Mugua treatment of emesis mainly include drug response, urea cycle, calcium ion response, arginine biosynthetic process, and positive regulation of NO biosynthetic process. The target proteins are mainly involved in the construction of mitochondrial matrix, mitochondrial inner membrane, membrane rafts, cytoskeletal fibers, and axons in terms of CCs. In terms of MFs, they are mainly involved in regulating flavin adenine dinucleotide binding, electron carrier activity, acyl-CoA activity, catalytic activity, and oxidoreductase activity. The enriched KEGG pathways were mainly involved in the degradation of valine, leucine, and isoleucine, fatty acid degradation, amino acid biosynthesis, fatty acid metabolism, and PPAR signaling pathway.

GO enrichment analysis revealed that inflammatory responses were involved in the treatment of rheumatoid arthritis, diarrhea, and edema by Mugua, and KEGG pathway enrichment revealed that inflammation-related pathways, such as rheumatoid arthritis pathway, TNF signaling pathway, NF-κB signaling pathway, PI3K-Akt signaling pathway, and PPAR signaling pathway, were involved. This finding also indicates that the traditional efficacy of Mugua is closely related to inflammation.

### 3.6. Construction of “component-target-disease-effect” network of Mugua

**Figure 8.**
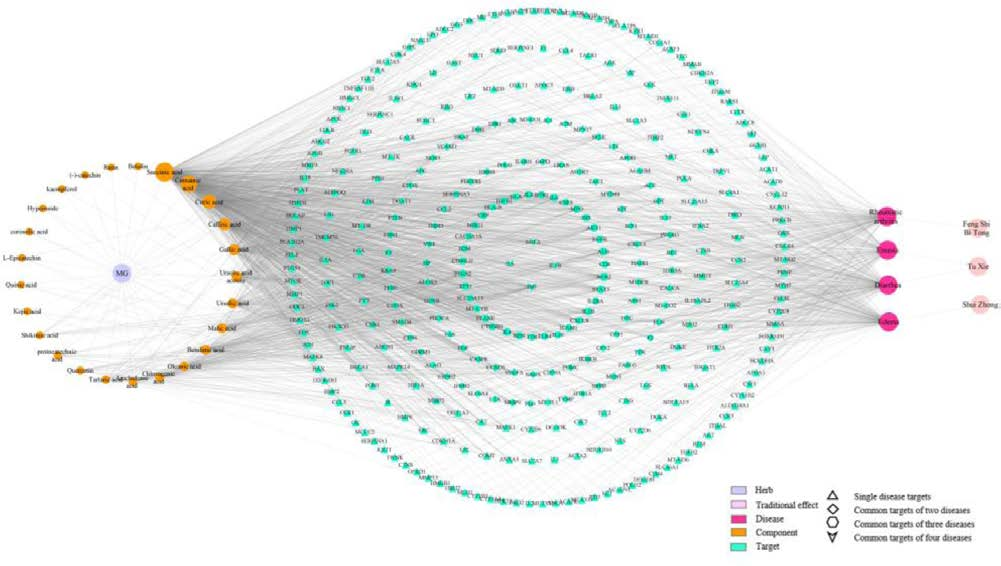
The “component-target-disease-effect” network of Mugua.

The relationships between components, targets, diseases, and effects were visualized, and the “component-target-disease-effect” network relationship diagram of Mugua was constructed in Cytoscape software based on the corresponding relationships between nodes (Fig. 8). The purple nodes in the diagram represent Mugua, the orange nodes represent active components, the blue nodes represent targets, the red nodes represent modern diseases corresponding to traditional efficacy, and the pink nodes represent traditional efficacy. Different shapes represent the shared targets of different diseases; the larger the degree value is, the larger the circle, indicating greater importance in the network. The network has 375 nodes and 2109 edges. Topological analysis of the network revealed that the 9 components with the highest degree values were succinic acid (degree value = 360), cinnamic acid (degree value = 273), citric acid (degree value = 200), caffeic acid (degree value = 144), gallic acid (degree value = 109), ursolic acid (degree value = 79), malic acid (degree value = 73), betulinic acid (degree value = 66), and oleanolic acid (degree value = 51), indicating that they are the key active components that exert these effects. The network diagram reveals the multi-component and multi-target synergistic mechanism by which Mugua exerts its traditional efficacy.

### 3.7. Molecular docking verification

**Figure 9.**
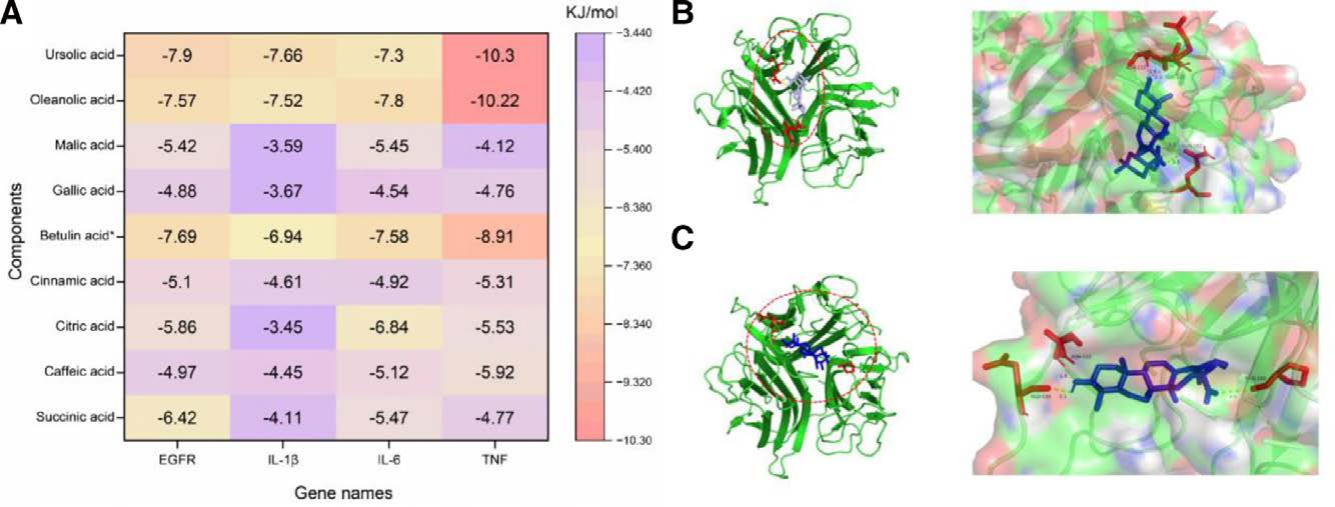
Molecular docking of the core compound-target pair of Mugua. (A) Heat map of estimated binding energy. Molecular docking of oleanolic acid-TNF pair (B) and ursolic acid-TNF pair (C).

Molecular docking verification was performed on the shared core targets of the 4 diseases and the top 9 components. A binding energy less than zero indicates that the ligand component can spontaneously bind to the receptor target protein. A binding energy less than −5.0 kJ/mol indicates good binding. Figure 9A shows that the 9 active components can all bind spontaneously to the 4 receptor target proteins, and oleanolic acid, ursolic acid, and betulinic acid have good binding with all 4 targets. The results of molecular docking with binding energies less than −10.00 kJ/mol were visually processed using PyMol software, and the results are shown in Figure 9B and C. The binding results of TNF and oleanolic acid show that oleanolic acid has a hydrogen bond with the amino acid residues ASN-112 and GLU-110, and 2 hydrogen bonds with GLN-102. The binding results of TNF and ursolic acid show that ursolic acid has a hydrogen bond with the amino acid residues ASN-112, GLU-110, and PRO-100. The molecular docking results show that Mugua exerts its traditional efficacy by intervening in inflammatory targets through key components.

### 3.8. In vitro anti-inflammatory effect of the active components of Mugua

The results of the in vitro anti-inflammatory experiment of the active components of Mugua are shown in Table 4. At a concentration of 50 μM, citric acid, succinic acid, and betulinic acid significantly reduced the level of IL-6 in the supernatant of BV-2 cells after LPS treatment; malic acid, cinnamic acid, caffeic acid, succinic acid, and citric acid significantly reduced the level of IL-1β in the cell supernatant; oleanolic acid, ursolic acid, cinnamic acid, citric acid, gallic acid, succinic acid, and betulinic acid significantly reduced the level of TNF-α in the cell supernatant; and malic acid, cinnamic acid, caffeic acid, lemon acid, gallic acid, and succinic acid significantly reduced the content of NO in the cell supernatant. The experimental results showed that all 9 components had strong anti-inflammatory activities at this concentration.

**Table 4:** The in vitro anti-inflammatory effects of the active components in Mugua (pg/mL, $\bar{x}\pm \;SD$, n = 3).

Group	IL-6	IL-1β	TNF-α	NO
Control	50.65 ± 6.33	62.71 ± 0.45	350.98 ± 54.32	8.72 ± 1.73
LPS	64.18 ± 3.30*	84.91 ± 4.40*	720.56 ± 51.07*	17.51 ± 1.30*
Oleanolic acid	59.05 ± 3.26	74.03 ± 2.62	531.67 ± 55.69**	16.19 ± 0.31
Ursolic acid	61.96 ± 2.92	74.03 ± 2.08	503.89 ± 37.47**	16.63 ± 0.54
Malic acid	55.73 ± 2.18	62.92 ± 3.00**	609.44 ± 30.68	12.67 ± 0.54**
Cinnamic acid	53.15 ± 4.65	65.97 ± 4.70**	584.44 ± 37.47**	12.67 ± 0.54**
Caffeic acid	59.84 ± 1.98	67.42 ± 1.70**	665.42 ± 33.20	11.79 ± 0.31**
Citric acid	42.68 ± 1.10**	64.47 ± 9.56**	573.75 ± 31.04**	12.99 ± 0.58**
Gallic acid	54.32 ± 1.85	72.73 ± 4.46	575.83 ± 25.17**	12.28 ± 0.58**
Succinic acid	49.97 ± 6.39**	67.27 ± 4.25**	337.29 ± 51.12**	13.94 ± 0.88**
Betulinic acid	43.72 ± 2.88**	66.65 ± 3.10**	249.79 ± 20.62**	12.99 ± 0.58**

Abbreviation: LPS = lipopolysaccharide.

* Compared with the blank control group, *P* < .05.

** Compared with the LPS control group, *P* < .05.

## 4. Discussion

The “Compendium of Materia Medica” records that Mugua is sour in taste, warm in nature, harmonizes the stomach and soothes the liver, activates muscles and tendons, and relaxes the meridians. Modern studies have shown that Mugua contains triterpenoids, flavonoids, and organic acid compounds, which have analgesic, anti-inflammatory, antimicrobial, hepatoprotective, and antitumor effects.^[1]^

Flavonoids are active components in the treatment of rheumatoid arthritis in Chinese herbal medicine, and their mechanisms of action may be related to their anti-inflammatory and antioxidant effects.^[9]^ Quercetin can inhibit the NF-κB pathway, thereby reducing the release of MMP-13, reducing the apoptosis of chondrocytes, alleviating tissue inflammation, and protecting cartilage tissue.^[10]^ β-Sitosterol can reduce the expression of COX-2 and inflammatory factors (TNF-α, IL-1β, and IL-6), playing an anti-inflammatory role.^[11]^ Oleanolic acid promotes lymphocyte proliferation, which can regulate the immune system and inhibit inflammation.^[12]^ 3-Acetylursolic acid in Mugua can inhibit the expression of MMP-3, MMP-1, and MMP-13, and inhibit the secretion of the MMP-3 protein in vivo to protect bone cells and alleviate rheumatoid arthritis.^[13]^ Studies have shown that the NF-κB signaling pathway plays a very important role in BPes such as the immune response and cellular inflammatory response. When the body is stimulated by external factors, IκBα in the NF-κB/IκBα complex is activated and phosphorylated, causing NF-κB to be released and enter the cell nucleus, inducing an inflammatory response.^[14]^ The TNF signaling pathway is an important pathway that can promote the expression of chemokines, growth factors, IL-1β, and TNF-α, and amplify the body’s inflammatory and immune responses.^[15]^ In summary, Mugua may act on key targets such as TNF and IL-6 through components such as β-sitosterol, oleanolic acid, 3-acetylursolic acid, and quercetin, and subsequently regulate the TNF signaling pathway and NF-κB signaling pathway to treat rheumatoid arthritis.

Studies have shown that quercetin can effectively treat diarrhea and can repair epithelial cells and has certain antimicrobial capabilities.^[16]^ β-Sitosterol has anti-inflammatory and intestinal contraction-relaxing effects,^[11]^ thereby preventing diarrhea. Chen et al reported that Mugua extract can treat diarrhea induced by intestinal toxins in rats, and its mechanism of action may involve blocking the binding of the intestinal toxin B subunit and ganglioside GM1.^[17]^ Studies have shown that the total triterpenoids of Mugua can inhibit the production of pro-inflammatory factors and upregulate the expression of intestinal mucosal barrier protective factors to treat diarrhea.^[18]^ The PI3K-Akt signaling pathway can regulate the expression of TNF-α, IL-1β, and IL-6, reduce cell apoptosis, and alleviate intestinal mucosal edema, playing a role in relieving diarrhea.^[19]^ Mugua may act on targets such as quercetin, β-sitosterol, and triterpenoid components, affecting the PI3K-Akt signaling pathway and playing a role in treating diarrhea.

Studies have shown that quercetin can act on related proteins such as IL-1β and IL-6, and downregulate the expression of inflammatory factors, thereby alleviating inflammation and inhibiting edema.^[20]^ PPI network analysis and KEGG pathway analysis revealed that the treatment of edema with Mugua is closely related to TNF-α, VEGF, and other proteins, as well as PI3K/AKT signaling pathway and HIF-1 pathway. The initiating factor TNF-α may induce the production of endogenous NO and PGE2, which participate in the pathological process of post-traumatic brain edema.^[21]^ TNF-α can also damage the blood-brain barrier through various channels, upregulate the expression of AQP4, and aggravate the condition of brain edema.^[22]^ VEGF is a vascular endothelial growth factor that can regulate the migration behavior of endothelial cells.^[23]^ VEGF can change the function of endothelial tight junction proteins, increase vascular permeability, and aggravate capillary leakage and edema.^[24]^ Studies have shown that the expression of HIF-1α around the brain hemorrhage foci is significantly increased, which is closely related to brain edema. HIF-1α is an important target for the treatment of brain hemorrhage.^[25]^ Kim et al reported that in the process of vascular edema formation, PI3K/AKT may be a common downstream molecule of the TRPC3 and ETB receptor signal transduction pathways, and the PI3K/AKT signaling pathway may be an important therapeutic target pathway for vascular edema.^[26]^ It is speculated that Mugua acts on targets such as TNF-α and VEGF, regulates the PI3K/AKT and HIF-1 signaling pathways, and plays a role in treating edema.

Oral chemotherapy drugs can cause gastrointestinal mucosal damage, leading to nausea and emesis. The pathological process is related to oxidative stress and the activation of related pathways. The drug response can cause gastric mucosal damage, and triterpenoid compounds in Mugua can increase gastric juice secretion, thereby restoring the defensive barrier function of the gastric mucosa and playing a role in treating gastric mucosal damage.^[27]^ Zhong et al found that Ca^2+^ flows into cells through L-type Ca^2+^ channels, activating the endoplasmic reticulum receptor RyRs to release more Ca^2+^, and causing a sharp increase in intracellular Ca^2+^ levels, thereby activating the ERK1/2 signaling pathway and causing emesis.^[28]^ Therefore, Mugua may regulate intracellular Ca^2+^ levels, reduce the release of Ca^2+^ from the sarcoplasmic reticulum and endoplasmic reticulum, inhibit the activation of the ERK1/2 pathway and related proteins, and stop emesis. In addition, the antispasmodic soup has a certain therapeutic effect on chemotherapy-induced emesis in the upper gastrointestinal tract, and the mechanism of improving emesis may be related to amino acid metabolism and fatty acid metabolism disorders.^[29]^ It is speculated that Mugua may regulate intracellular Ca^2+^ levels, improve amino acid metabolism, and fatty acid metabolism through triterpenoid components, playing a role in treating emesis.

## 5. Conclusion

In summary, this work used network pharmacology to preliminarily study the mechanism by which Mugua exerts its effects on soothing muscles and activating collaterals, harmonizing the stomach, and transforming dampness in treating rheumatoid arthritis, diarrhea, edema, and emesis. The shared targets of the 4 diseases are all related to inflammation. GO enrichment and KEGG pathway enrichment results showed that the traditional efficacy of Mugua was closely related to inflammatory responses. The network diagram identified 9 key active components of Mugua, revealing the multi-component and multi-target synergistic mechanism by which Mugua exerts its traditional efficacy. Molecular docking results and in vitro anti-inflammatory activity experiments showed that Mugua exerts its traditional efficacy by intervening in inflammatory targets through key components. This study lays the foundation for in-depth research on the material basis and mechanism of action of Mugua and provides a scientific basis for its clinical application.

## Acknowledgments

The corresponding author thank Mr Zhao Yang for his support of this work. The authors are thankful to the Natural Science Foundation of Shaanxi Province (2022SF-221), and University-level Research Project of Shaanxi University of Chinese Medicine (2023GP24).

## Author contributions

**Conceptualization:** Shijun Yue.

**Data curation:** Yonggang Wu.

**Formal analysis:** Yonggang Wu.

**Funding acquisition:** Shijun Yue.

**Investigation:** Yonggang Wu.

**Methodology:** Yonggang Wu.

**Project administration:** Shijun Yue.

**Resources:** Shijun Yue.

**Supervision:** Shijun Yue.

**Validation:** Yonggang Wu.

**Writing – original draft:** Yonggang Wu, Shijun Yue.

**Writing – review & editing:** Yonggang Wu, Shijun Yue.
